# Supplementary material for: IDV Typer: An Automated Tool for Lineage Typing of Influenza D Viruses Based on Return Time Distribution
Source: Viruses. 2024 Feb 28;16(3):373. doi: 10.3390/v16030373 (PMC10976072; doi:10.3390/v16030373)
Supplement: Supplementary file 1 [file viruses-16-00373-s001.zip › viruses-2826764-supplementary.pdf]

Table S1. Reference dataset.

Sr. No	Accession ID	Lineage	Reference (Pubmed ID)
1	NC_036618	D/OK	23408893
2	KM015501	D/OK	35909676
3	KT592522	D/OK	31801277
4	OM468246	D/OK	35216016
5	MT511460	D/OK	37491320
6	OM468247	D/OK	35216016
7	OM468296	D/OK	35216016
8	MK965261	D/OK	35909676
9	MT511488	D/OK	37491320
10	MN123883	D/660	37491320
11	MW079473	D/660	33771071
12	MW632181	D/660	33771071
13	MW632188	D/660	33771071
14	MT511412	D/660	37491320
15	OM468233	D/660	35216016
16	ON166840	D/660	36533151
17	LC128433	D/Yama2016	35909676
18	LC565476	D/Yama2016	35909676
19	LC494108	D/Yama2019	35909676
20	ON415266	D/Yama2019	35909676
21	MW020308	D/CA2019	33771071
22	MW020322	D/CA2019	33771071
23	LN559126	D/France2012	32611750

Table S2. True positive dataset

Sr. No	Accession ID	Lineage	Reference (Pubmed ID)
1	KM015508	D/OK	35909676
2	KM015494	D/OK	35909676
3	KT581412	D/OK	37491320
4	KT581418	D/OK	37491320
5	KU171129	D/OK	37491320
6	KX768827	D/OK	31801277
7	KX768834	D/OK	31801277
8	LC522354	D/OK	32102883
9	MT636473	D/OK	35909676
10	MW632174	D/OK	33771071
11	MZ264979	D/OK	Not available
12	MK131035	D/OK	31801277
13	MN165188	D/OK	31801277
14	MN165209	D/OK	31801277
15	MN165257	D/OK	31801277
16	MN123953	D/OK	31801277
17	MT349627	D/OK	36533151
18	MW079478	D/OK	35909676
19	KT592526	D/OK	31801277

20	MN123939	D/OK	31801277
21	MN165216	D/OK	31801277
22	OM468237	D/OK	35216016
23	OM468238	D/OK	35216016
24	OM468239	D/OK	35216016
25	OM468240	D/OK	35216016
26	OM468241	D/OK	35216016
27	OM468242	D/OK	35216016
28	OM468243	D/OK	35216016
29	OM468244	D/OK	35216016
30	OM468245	D/OK	35216016
31	MN165202	D/OK	31801277
32	MK101121	D/OK	35909676
33	MN165195	D/OK	31801277
34	KF425669	D/OK	35909676
35	MG720235	D/OK	35909676
36	MH315965	D/OK	35909676
37	MH315966	D/OK	35909676
38	MH315967	D/OK	35909676
39	MH315968	D/OK	35909676
40	MH315969	D/OK	35909676
41	MH315970	D/OK	35909676
42	MK965258	D/OK	31801277
43	MK965262	D/OK	35909676
44	MK965263	D/OK	35909676
45	MK965264	D/OK	35909676
46	MK965268	D/OK	35216016
47	MK965269	D/OK	35216016
48	MK965270	D/OK	35216016
49	MK965271	D/OK	35909676
50	MK965273	D/OK	35216016
51	MK965278	D/OK	35909676
52	MT511357	D/OK	37491320
53	MT511364	D/OK	37491320
54	MT511377	D/OK	37491320
55	MT511384	D/OK	37491320
56	MT511398	D/OK	37491320
57	MT511405	D/OK	37491320
58	MT511426	D/OK	37491320
59	MT511433	D/OK	37491320
60	MT511440	D/OK	37491320
61	MT511453	D/OK	37491320
62	MT511467	D/OK	37491320
63	MT511474	D/OK	37491320
64	MT511481	D/OK	37491320
65	MT511495	D/OK	37491320
66	MT511502	D/OK	37491320
67	MT511515	D/OK	37491320
68	MT511522	D/OK	37491320
69	MT511536	D/OK	37491320
70	MT511543	D/OK	37491320

71	OM468248	D/OK	35216016
72	MK965259	D/OK	31801277
73	MK965265	D/OK	35216016
74	MK965266	D/OK	35216016
75	OK513025	D/OK	35537488
76	MT349564	D/OK	36533151
77	MK131026	D/OK	37491320
78	MN165223	D/OK	31801277
79	MK965277	D/OK	37491320
80	KM392492	D/660	35909676
81	KT581416	D/660	37491320
82	KT581417	D/660	37491320
83	KU171126	D/660	35909676
84	KU171127	D/660	35909676
85	KU171128	D/660	35909676
86	MN123959	D/660	37491320
87	MN123890	D/660	37491320
88	MN123925	D/660	37491320
89	MN123897	D/660	37491320
90	MN123946	D/660	37491320
91	MN123911	D/660	37491320
92	MT349516	D/660	36533151
93	MT349523	D/660	36533151
94	MT349530	D/660	36533151
95	MT349599	D/660	36533151
96	MT349606	D/660	36533151
97	MT349647	D/660	36533151
98	MT349654	D/660	36533151
99	MN123869	D/660	37491320
100	MW079472	D/660	37491320
101	MW079475	D/660	37491320
102	MW079476	D/660	37491320
103	MW079474	D/660	37491320
104	MW079477	D/660	37491320
105	OM468234	D/660	35216016
106	OM468235	D/660	35216016
107	OM468236	D/660	35216016
108	MT246289	D/660	37491320
109	MT246269	D/660	37491320
110	MK054184	D/660	35909676
111	MN173611	D/660	31801277
112	MT511336	D/660	37491320
113	MT511391	D/660	37491320
114	MT511419	D/660	37491320
115	MT511529	D/660	37491320
116	MT246282	D/660	37491320
117	MT246276	D/660	37491320
118	LC270268	D/Yama2016	35909676
119	LC318668	D/Yama2016	35909676
120	LC565477	D/Yama2019	35909676
121	MW020315	D/CA2019	33771071

122	ON038774	D/OK	36533151
123	ON038776	D/OK	36533151
124	ON038778	D/OK	36533151
125	ON038779	D/OK	36533151
126	ON038782	D/OK	36533151
127	ON038794	D/OK	36533151
128	ON038934	D/OK	36533151
129	ON038935	D/OK	36533151
130	OP353622	D/OK	36103901
131	KT592533	D/OK	37491320
132	ON166834	D/660	36533151
133	ON166835	D/660	36533151
134	ON166836	D/660	36533151
135	ON166837	D/660	36533151
136	ON166838	D/660	36533151
137	ON166839	D/660	36533151
138	ON166842	D/660	36533151
139	ON166843	D/660	36533151
140	OP474074	D/660	37347825
141	OP020136	D/Yama2016	37491320
142	OP020135	D/Yama2019	37491320
143	OP353623	D/Yama2019	36103901

Table S3. True Negative dataset

Sr. No	Accession ID	Gene	Virus Name
1	KP417469	PA	Influenza A Virus
2	MW220337	PA	Influenza A Virus
3	OR762342	PA	Influenza A Virus
4	KM654714	PB1	Influenza A Virus
5	KP286559	PB1	Influenza A Virus
6	EF124030	PB1	Influenza A Virus
7	FJ805464	PB2	Influenza A Virus
8	DQ992569	PB2	Influenza A Virus
9	MZ717369	PB2	Influenza A Virus
10	MW547645	NP	Influenza A Virus
11	KX351451	PB1	Influenza B Virus
12	MZ951444	PB1	Influenza B Virus
13	MK961711	PB2	Influenza B Virus
14	KM654702	PB2	Influenza B Virus
15	KM654724	PB2	Influenza B Virus
16	CY182406	PA	Influenza B Virus
17	MH684318	PA	Influenza B Virus
18	KM654766	PA	Influenza B Virus
19	JQ396208	HA	Influenza B Virus
20	KX615582	HA	Influenza B Virus
21	MH726977	HA	Influenza B Virus
22	MF195765	HA	Influenza B Virus
23	MH684330	NP	Influenza B Virus
24	KX615311	NP	Influenza B Virus

25	OQ997395	NP	Influenza B Virus
26	JX513007	NP	Influenza B Virus
27	MH348116	HE	Influenza C Virus
28	GQ853452	HE	Influenza C Virus
29	LC122648	HE	Influenza C Virus
30	KT232085	HE	Influenza C Virus
31	KM504281	NP	Influenza C Virus
32	FR671423	NP	Influenza C Virus
33	ON038921	PB2	Influenza D Virus
34	MT349586	PB2	Influenza D Virus
35	MK965340	P3	Influenza D Virus
36	KX424578	NP	Isavirus
37	KX823915	NP	Isavirus
38	GU830905	NP	Isavirus
39	AF287950	PB1	Isavirus
40	KU587559	PA	Isavirus
41	KU587567	PA	Isavirus
42	KU587575	PA	Isavirus
43	MN053829	PB2	Quaranjavirus
44	MZ502308	PB2	Quaranjavirus
45	MW256693	NP	Quaranjavirus
46	ON160025	NP	Quaranjavirus
47	MW256684	HA	Quaranjavirus
48	MH688515	PA	Thogotovirus
49	MT628436	PA	Thogotovirus
50	AF006073	PA	Thogotovirus
